# Supplementary material for: Ultrahigh-efficient material informatics inverse design of thermal metamaterials for visible-infrared-compatible camouflage
Source: Nat Commun. 2023 Aug 4;14:4694. doi: 10.1038/s41467-023-40350-6 (PMC10403604; doi:10.1038/s41467-023-40350-6)
Supplement: Supplementary file 1 — Supplementary Information [file 41467_2023_40350_MOESM1_ESM.pdf]

Supplementary Information

Ultrahigh-efficient material informatics inverse design of thermal metamaterials for visible-infrared-compatible camouflage

Wang Xi[1,a], Yun-Jo Lee[2,a], Shilv Yu[1], Zihe Chen[1], Junichiro Shiomi[3], Sun-Kyung Kim[2,*], Run Hu[1,*]

[1]*School of Energy and Power Engineering, Huazhong University of Science and Technology, Wuhan 430074, China*

[2]*Department of Applied Physics, Kyung Hee University, 1732 Deogyeong-daero, Giheung-gu, Yongin-Si, Gyeonggi-do 17104, Republic of Korea*

[3]*Department of Mechanical Engineering, University of Tokyo, 7-3-1 Hongo, Bunkyo, Tokyo 113-8656, Japan*

[a]*These authors contributed equally.*

*Corresponding author: R.H. (hurun@hust.edu.cn); S.K.K. (sunkim@khu.ac.kr)

Supplementary Note 1

Structure Configuration and Design Space Setup

In our thermal metamaterial setup, we choose multilayer structure pattern to facilitate the design, optimization and fabrication. To enable the structure optimization with best performance for visible-infrared-compatible camouflage, the design space should be large enough while the computation cost should also be taken into consideration, and thus a tradeoff is a must. Suppose that the thermal metamaterials consist of m layers, and each layer has n choices including the materials and thickness, resulting in the total design space as much as n^m. For example, for a five-layer structure (m=5), each layer thickness ranges from 20 nm to 1 μm with a spacing of 10 nm with two kinds of material choices (n=99×2). Therefore, the design space is $(99\times2)^5 = 3.04\times10^{11}$, which may be too large to solve and will consume a huge amount of time.

To make it solvable, we shrink the design space by three steps. Firstly, the whole structure is divided into two parts, which can be used to tune the VIS and IR emissivity respectively. A single topmost ZnS layer with tunable thickness is reserved to achieve difference colors[1], while the rest part corresponds to IR camouflage. After preliminary

study, the thickness range of the topmost ZnS sublayer is set as 20~600 nm with a spacing of 10 nm, resulting in 59 choices. Secondly, the thickness of remaining sublayers for IR camouflage is fixed at 200 nm to form Ge/ZnS pair, whose optical path is approximate to the quarter of target wavelength in the atmosphere window to induce low emissivity. Lastly, the number of sublayers for IR camouflage is set to 10, which can cause the design space in an order of 10^4 ($59\times2^{10}=6\times10^4$) and makes calculation time-friendly. It should be noted that our structure configuration is only expressive for the layered pattern. Structures in other patterns (such as grating pattern, nanorod pattern, etc.), which may possibly exhibit more colors or better infrared camouflage performance, are not included in our design roadmap. Nevertheless, the roadmap offers a universal design approach, which can also be used for other structure configuration optimization after necessary modifications including structure pattern, design space and simulation method. More discussions are presented in Supplementary Note 2.

Supplementary Note 2

Color Simulation and Color Difference Characterization

The color of our structure derives from selectively reflected sunlight in visible band (0.38-0.78μm). Color is related to three parameters, including the illuminant, the surface of the structure, and the observer. When the light from illuminant shines on the surface of the opaque structure, part of it will be absorbed while others will be reflected. The reflected part will be captured by human eyes and stimulates photoreceptors to produce electrical signals, which forms our perception of color. The CIE (International Committee on illumination) uses the tristimulus values to describe this procedure, which can be described as follow[2]:

$$X=\mathrm{k}\int_{380}^{780} s(\lambda)R(\lambda)\bar{x}(\lambda)\,d\lambda \qquad \text{S.(19)}$$

$$Y=\mathrm{k}\int_{380}^{780} s(\lambda)R(\lambda)\bar{y}(\lambda)\,d\lambda \qquad \text{S.(20)}$$

$$Z=\mathrm{k}\int_{380}^{780} s(\lambda)R(\lambda)\bar{z}(\lambda)\,d\lambda \qquad \text{S.(21)}$$

$$\mathrm{k}=100\Big/\int_{380}^{780} s(\lambda)\bar{y}(\lambda)\,d\lambda \qquad \text{S.(22)}$$

where *X, Y, Z* is the tristimulus values, *s(λ)* is the spectral power distribution of the

illuminant, which is set to be the standard D65 illumination in our case to represent the outdoor daytime sunlight. *R(λ)* is the reflectivity of the structure, $\bar{x}(\lambda)$, $\bar{y}(\lambda)$, and $\bar{z}(\lambda)$ are the color matching function, which are the spectral tristimulus values under standard chroma. *k* is the normalization parameter. Once the reflectivity of the structure is obtained, the color can be simulated quantitatively.

To measure the color difference, several color spaces have been put forward, such as CIE-Lab, CIE-LCH, and CMC (l: c). CIE-Lab color space has three parameters L^*, a^*, and b^*. L^* is used to represent lightness while a^* and b^* are used to represent chromaticity. These parameters can be transformed from the three tristimulus value *X, Y, Z* in the CIE-XYZ color space numerically as:

$$L^* = 116 f\left(Y/Y_0\right) - 16 \qquad \text{S.(23)}$$

$$a^* = 500\left[f\left(X/X_0\right) - f\left(Y/Y_0\right)\right] \qquad \text{S.(24)}$$

$$b^* = 200\left[f\left(Y/Y_0\right) - f\left(Z/Z_0\right)\right] \qquad \text{S.(25)}$$

$$f(t) = \begin{cases} t^{\frac{1}{3}}, & t > \left(\dfrac{24}{116}\right)^3 \\ \left(\dfrac{841}{108}\right)t + \dfrac{16}{116}, & t \le \left(\dfrac{24}{116}\right)^3 \end{cases} \qquad \text{S.(26)}$$

where X_0, Y_0, Z_0 are the tristimulus values corresponding to color white. Similarly, CIE-LCH uses lightness ($L=L^*$), chroma ($C=\sqrt{a^{*2}+b^{*2}}$), and hue ($H = \arctan(b/a)$) to describe colors, which can be expressed by combination of the L^*, a^*, and b^*, respectively. Compared to these color spaces, CMC (l: c) uses two weight factors to amend the lightness difference and chroma difference, which make it more close to human sensation of color difference. The color difference of two target color can be calculated as follow:

$$\Delta E_{CMC(l:c)} = \sqrt{\left(\frac{L_1 - L_2}{l \cdot S_L}\right)^2 + \left(\frac{C_1 - C_2}{c \cdot S_C}\right)^2 + \left(\frac{H_1 - H_2}{S_H}\right)^2} \qquad \text{S.(27)}$$

$$S_L = \begin{cases} 0.040975 L_1 / \left(1 + 0.01765 L_1\right), & L_1 \ge 16 \\ 0.511, & L_1 < 16 \end{cases} \qquad \text{S.(28)}$$

$$S_C = \frac{0.0638C_1}{1+0.0131C_1} + 0.638 \quad \text{S.(29)}$$

$$S_H = S_C\left(F \cdot T + 1 - F\right) \quad \text{S.(30)}$$

$$F = \sqrt{\frac{C_1^4}{C_1^4 + 1900}} \quad \text{S.(31)}$$

$$T = \begin{cases} 0.36 + \left|0.4\cos\left(h_1 + 35\right)\right|, & h_1 > 345^\circ \,||\, h_1 < 164^\circ \\ 0.56 + \left|0.2\cos\left(h_1 + 168\right)\right|, & 164^\circ \le h_1 \le 345^\circ \end{cases} \quad \text{S.(32)}$$

where L_1, C_1, H_1 are the lightness, chroma, hue of the target color, while L_2, C_2, H_2 are the counterparts of the compared color. l and c are the weight factor, which is set to be 2 and 1.

Supplementary Note 3

Expanding the Feasible Color Range

In our proposed structure, only a tunable ZnS layer is settled to demonstrate color for simplification. Therefore, the obtained color range is limited due to the relatively simple structure. In fact, there are many other structure patterns along with diverse materials which can be utilized to expand the color range. For example, we design a new topmost structure pattern to obtain color out of the current color range, whose structure diagram is shown in Supplementary Fig. 2a. The topmost periodic structure, whose period is P, consists of a 70-nm-thick Al square pattern with a side length of L, a 35-nm-thick Ge layer, and a 100-nm-thick Ag layer from top to bottom. The structure beneath the topmost periodic structure is one of our optimized structures. This new proposed structure pattern can be used to obtain colors which are not obtainable with the original single ZnS layer. For instance, we design three structures (α, β, γ) from the new proposed structure pattern and simulate their visible reflectance with Rigorous Coupled Wave Analysis (RCWA)[3-4] to obtain their colors. The visible reflectance and colors are plotted in Supplementary Fig. 2c with the structure parameters labeled. The colors of three structures (α, β, γ) are marked as diamonds in CIE 1931 color space compared with the color range of the layered structures, which is lined out by the purple dotted line, as shown in Supplementary Fig. 2b. It can be seen that the colors of our new

proposed structures lie beyond the purple dotted line, which indicates an expansion of color range. It should be noted that the proposed structure here is just a demo. In fact, the 35-nm-thick Ge layer and the 100-nm-thick Ag layer, which is fixed here for simplification, can also be tunable to broaden the color range. Moreover, grating, rods, rectangles, etc. can be designed as new structure patterns with more tunable parameters. It can be inferred that there are great chance to expand the color range after integrating more complicated structure pattern in our inverse design framework. Necessary modification including simulation method and design space should be made to make whole framework works well. Also, the calculation cost would be more expensive when more complicated structure is integrated. One should make tradeoff between the VIS-IR camouflage performance of the obtained structure and the computation feasibility of the optimization algorithm.

Supplementary Note 4

Comparison of the infrared camouflage performance between our optimized structure and other empirically designed structure

To demonstrate the superiority of optimization design over empirically design, an empirically designed structure in another research on VIS-IR camouflage[5] is taken as an example to compare with our optimized structure. In their design, the Ge/ZnS multilayer structure shown in the Supplementary Fig. 3a below is manually designed to fulfill a reflectance tuning in both visible and infrared bands. We reproduce the infrared reflectance of their proposed structure and evaluate its RTS with the same environment settings. The accuracy of our simulated reflectance is verified by comparing with the counterpart in their text, which is shown in the Supplementary Fig. 3b. The blue solid line which represents our reproduced IR reflectance matches well with the red dashed line which represents the counterpart in their text, indicating the accuracy of our reproduced reflectance. The empirically designed structure exhibits an RTS of 0.5027, while all of our optimized results exhibit RTS below 0.42, with the best one with an RTS of 0.3471. Therefore, it is proved that our optimized structures have better infrared camouflage performance.

Supplementary Note 5

The Angle Dependence of the Designed Structure's Radiation

Supplementary Fig. 4 demonstrates the simulated reflectance of four camouflage structures in different incident angle θ. Each horizontal line represents a reflectivity spectrum corresponding one incident angle, whose reflectivity is indicated by the right color bar. The corresponding colors under different θ are also shown in the left colorbar. The angular reflectivity in visible shown in the left side is angle-insensitive, which ensure a similar color appearance and a consistent and reliable visible camouflage effect in all observing directions. The right side of panels show a nearly consistent infrared reflectance with high reflectance in atmosphere window and low reflectance (high emittance) in other infrared waveband for radiative cooling without heat accumulation, which also guarantee a consistent thermal camouflage effect.

Supplementary Note 6

Comparison between other empirically designed structure and our structures

There are some existing works on vis-IR compatible camouflage, among which Zhu's work is the most representative one[6]. They empirically designed a coupled structure (shown in the Supplementary Fig. 5a below), which consisted of a Ge/ZnS multilayer and a Cu-ITO-Cu metasurface, to demonstrate multispectral camouflage for the visible, mid-infrared (MIR, 3–5 and 8–14 μm), lasers (1.55 and 10.6 μm) and microwave (8–12 GHz) bands. They overcame the challenge of several spectral requirements with one structure and achieved a satisfying compatible camouflage effect, which is quite difficult and requires expert intuition.

In contrast, our research proposes a material-informatics-based inverse design framework to efficiently design metamaterials with desired camouflage performance, which avoids the difficult and complicated empirical design process. It should be noted that although the target is set as VIS-IR compatible camouflage in our text as an example, the inverse design framework also applies to more complicated situation such as VIS-IR-laser-microwave-compatible camouflage after modifying the optimization

target, structure pattern, alternative materials, simulation method, and so on.

Our second advantage over Zhu's work is that our results demonstrate more colors and equally good IR camouflage effect with leaner structure. For verification, the colors obtained in Zhu's work has been marked as black square in the CIE 1931 color space (Supplementary Fig. 5c) below. It can be seen that these colors are included in the purple dashed line, which shows that the color range we obtained is larger. The IR reflectance of part of their stacked structure (only the part which contributes to VIS-IR camouflage) has been also reproduced with its RTS evaluated in the same ambient condition. The accuracy of the result is verified by comparing with the IR reflectance in their paper, which is shown in the Supplementary Fig. 5b below. The RTS is calculated to be 0.3479, which is on par with our optimized structure (RTS=0.3471). Nevertheless, their structure contains 11 alternating Ge/ZnS layers whose thicknesses are 0.721/0.982/0.721/0.559/0.234/0.438/0.206/0.438/0.552/1.18/0.701 μm (from top to bottom), which is more complicated and harder to manufacture compared to our 3-layer structure. In conclusion, our widely applicable inverse design framework can free us from empirical design with thresholds to explore better performance and more streamlined structures, which may provide reference and spark new ideas for multi-objective optimization beyond multi-spectral camouflage. The comparison between Zhu's work and ours are listed below in Table T1.

Table T1 Comparison between Zhu's structure and ours

	Zhu's structure[6]	Our structure
Design Method	Empirically Designed	Optimized Automatically
Number of Total Layers	11 layers	3 layers
Total thickness	6.732 μm	~2.340 μm

Color Range	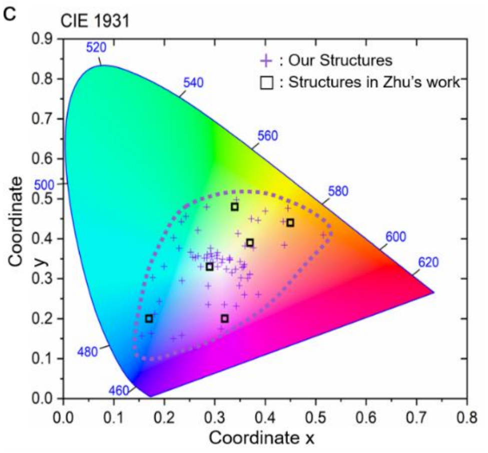<br>Color range obtained in Zhu's work (hollow square) and our work (purple dashed line)	
IR Camouflage Performance<br>(A smaller RTS means a better performance)	RTS=0.3479	RTS=0.3471

Supplementary References

1. D. Qi, et al., Effective strategy for visible-infrared compatible camouflage: surface graphical one-dimensional photonic crystal, *Opt. Lett.* **43** (21), 5323-5326 (2018).

2. W. Xi, et al., Colored radiative cooling: How to balance color display and radiative cooling performance, *Int. J. Therm. Sci.* **170**, 107172 (2021).

3. M. G. Moharam, T. K. Gaylord, Rigorous coupled-wave analysis of planar-grating diffraction, *J. Opt. Society of America* **71** (7), 811-818 (1981).

4. V. Liu, S. H. Fan, S-4: A free electromagnetic solver for layered periodic structures, *Comput. Phys. Commun.* **183** (10), 2233-2244 (2012).

5. Z. Deng, et al., Nanostructured Ge/ZnS Films for Multispectral Camouflage with Low Visibility and Low Thermal Emission, *ACS Appl. Nano Mater.* **5** (4), 5119-5127 (2022).

6. H. Zhu, et al., Multispectral camouflage for infrared, visible, lasers and microwave with radiative cooling, *Nat. Commun.* **12**, 1805 (2021).

Supplementary Figure

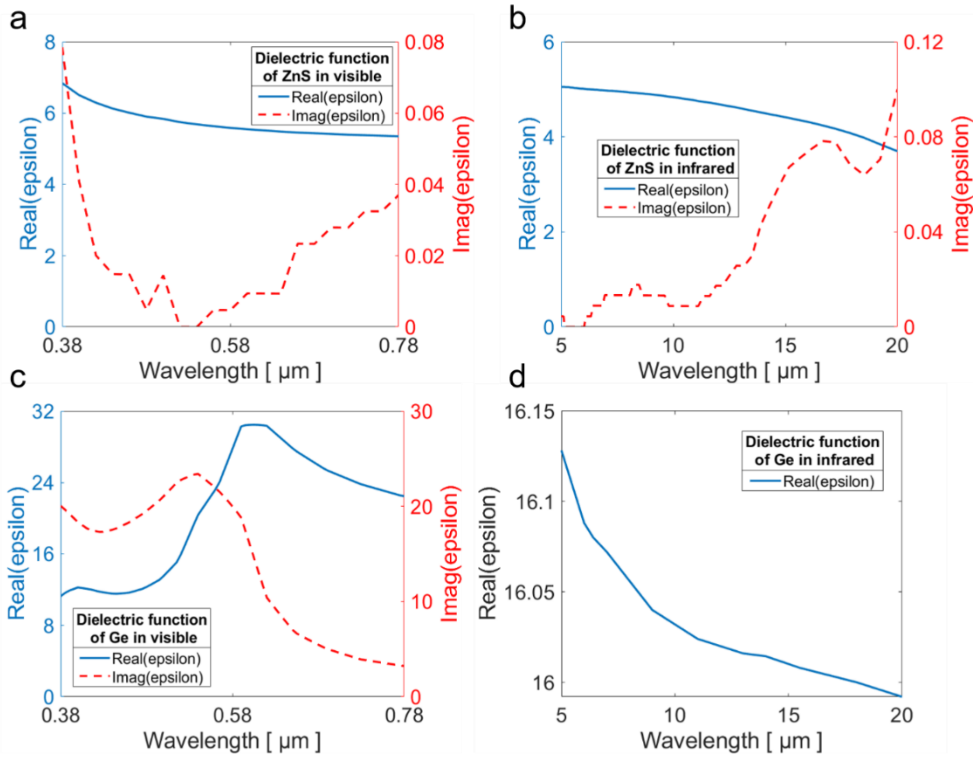

Supplementary Figure 1. **Dielectric function fitting. a** ZnS in visible waveband (0.38~0.78 μ m). **b** ZnS in infrared waveband (5~20 μm). **c** Ge in visible waveband (0.38~0.78 μm). **d** Ge in infrared waveband (5~20 μm).

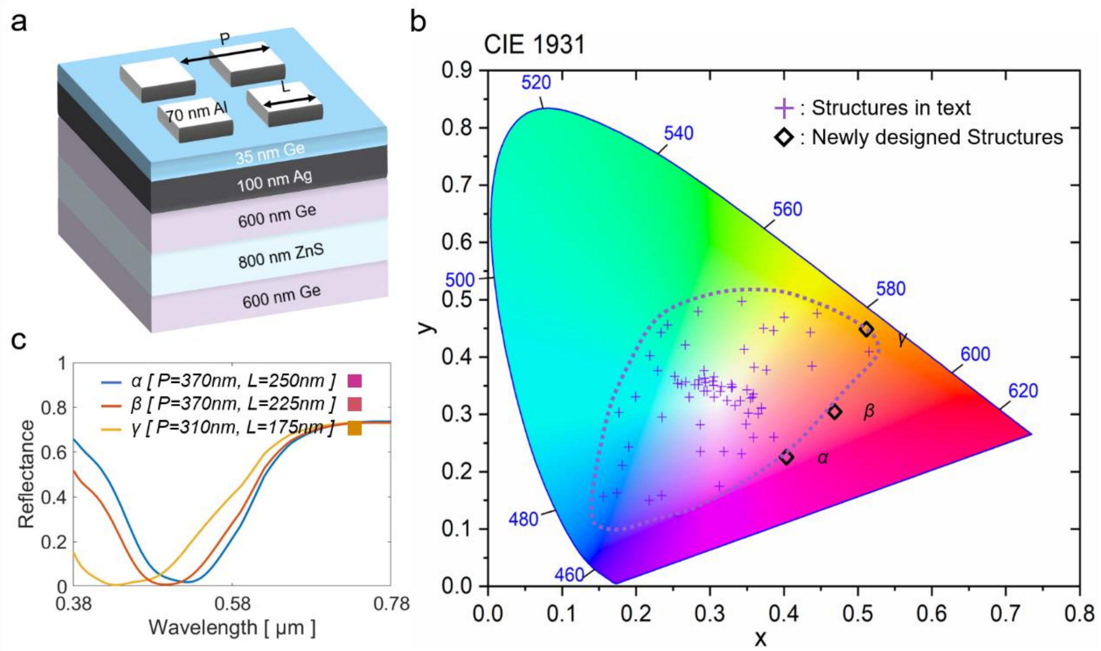

Supplementary Figure 2. **Metasurface pattern with expandable color range. a** Schematic of the new proposed structure pattern. **b** CIE 1931 color space. Purple crosses: layered structures designed by our inverse design algorithm; Purple dotted line: the color range of the layered structures; Black diamonds: the structures with new proposed pattern. **c** Visible Reflectance of the three new proposed structures. Corresponding structure parameters and the colors are labelled.

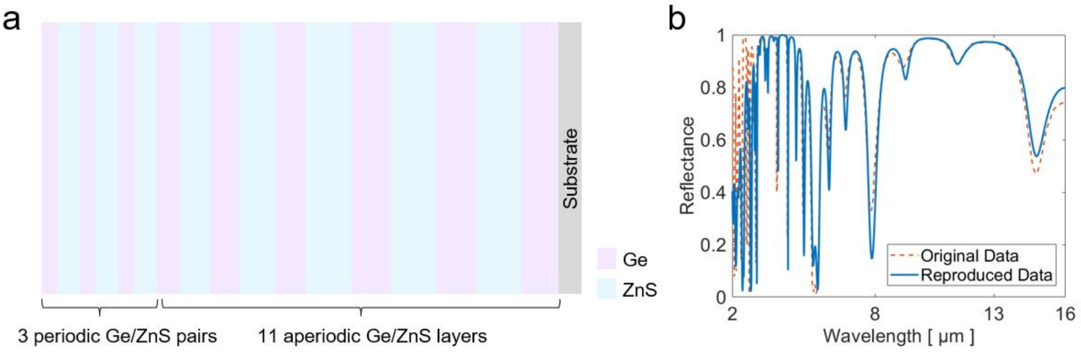

Supplementary Figure 3. **Comparison of the infrared camouflage performance between our optimized structure and an empirically designed structure in another research on VIS-IR camouflage. a** Schematic of the structure proposed in *ACS Appl. Nano Mater. 2022, 5, 5119−5127* [5]. **b** Reflectance spectra. Solid line: from our reproduced reflectance data; Dashed line: from original data.

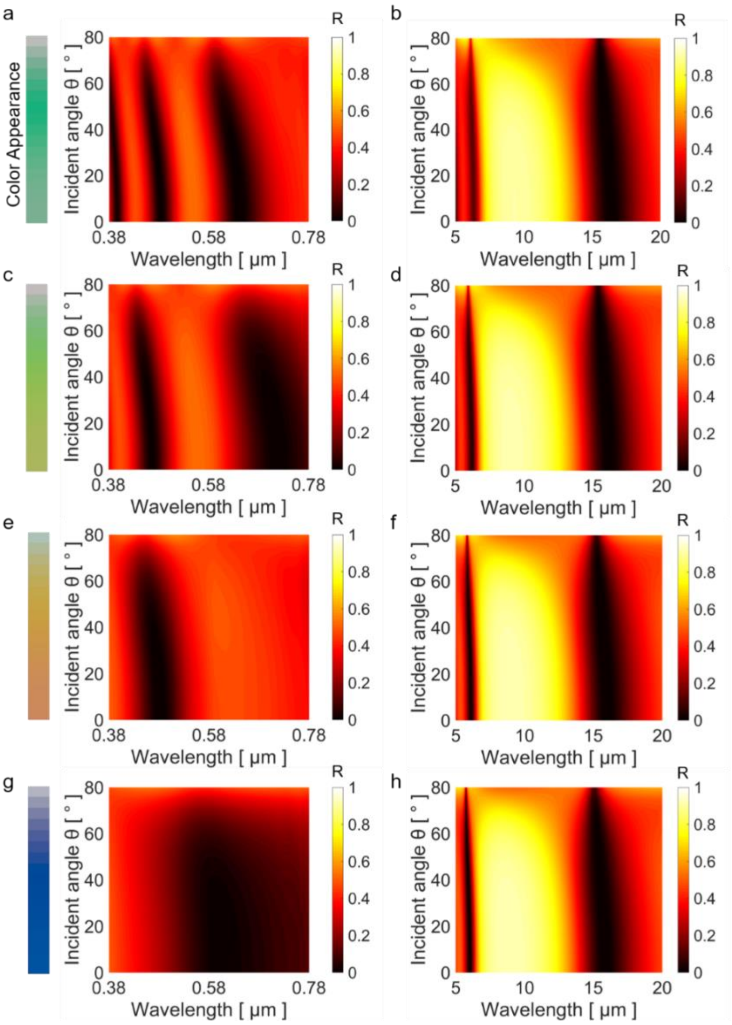

Supplementary Figure 4. **Simulated reflectivity spectra of four designed structures in visible(a, c, e, g) and IR(b, d, f, h) with their colors under different incident angles. a-b** Green color camouflage. **c-d** Yellow color camouflage. **e-f** Red color camouflage. **g-h** Blue color camouflage.

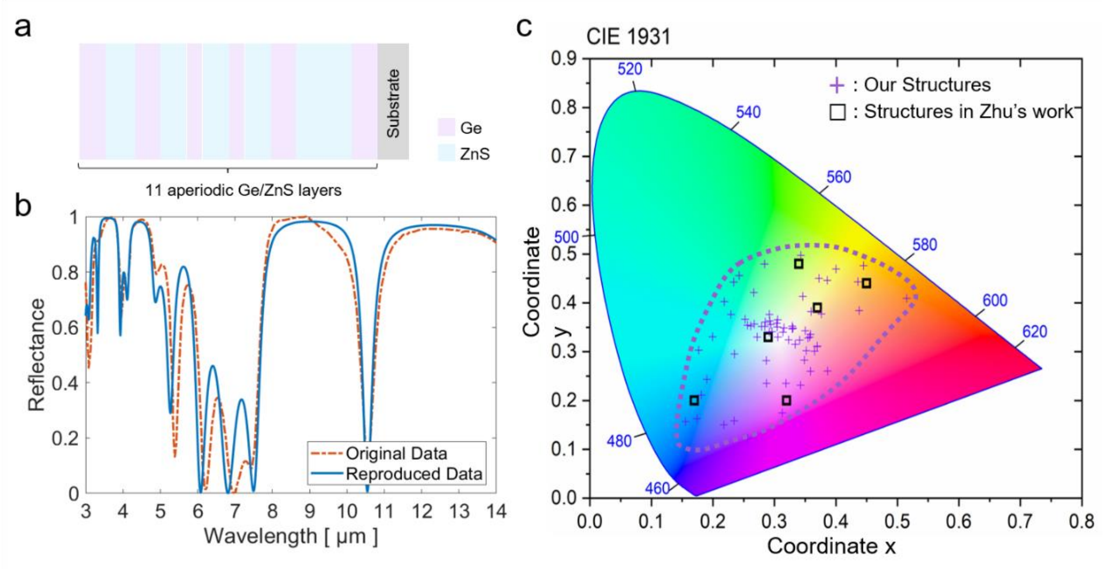

Supplementary Figure 5. **Comparison between other empirically designed structure and our structures. a** Schematic of the structure proposed in *Nat. Commun. 2021, 12, 1805* [6]. **b** Reflectance

spectra. Solid line: from our reproduced reflectance data; Dashed line: from original data. **c** CIE 1931 color space. Purple cross: structures designed by our inverse design algorithm; Purple dotted line: the color range of our structures; Black squares: the structures in *Nat. Commun. 2021, 12, 1805* [6].
