# Supplementary material for: Ultrahigh-efficient material informatics inverse design of thermal metamaterials for visible-infrared-compatible camouflage
Source: Nat Commun. 2023 Aug 4;14:4694. doi: 10.1038/s41467-023-40350-6 (PMC10403604; doi:10.1038/s41467-023-40350-6)
Supplement: Supplementary file 3 — Description of Additional Supplementary Files [file 41467_2023_40350_MOESM3_ESM.pdf]

Description of Additional Supplementary Files:

Supplementary Dataset 1: Experimentally measured visible reflectivity and infrared emissivity spectra for 8 samples.
